# Fruit Salad in the Lab: Comparing Botanical Species to Help Deciphering Fruit Primary Metabolism

**DOI:** 10.3389/fpls.2019.00836

**Published:** 2019-07-09

**Authors:** Léa Roch, Zhanwu Dai, Eric Gomès, Stéphane Bernillon, Jiaojiao Wang, Yves Gibon, Annick Moing

**Affiliations:** ^1^UMR1332 Biologie du Fruit et Pathologie, Centre INRA de Bordeaux, INRA, Université de Bordeaux, Bordeaux, France; ^2^UMR 1287 EGFV, INRA, Bordeaux Sciences Agro, Université de Bordeaux, Bordeaux, France; ^3^Plateforme Métabolome Bordeaux, CGFB, MetaboHUB-PHENOME, IBVM, Centre INRA de Bordeaux, Bordeaux, France

**Keywords:** amino acids, cross-species, fleshy fruit, inter-species, metabolism regulation, organic acids, primary metabolism, sugars

## Abstract

Although fleshy fruit species are economically important worldwide and crucial for human nutrition, the regulation of their fruit metabolism remains to be described finely. Fruit species differ in the origin of the tissue constituting the flesh, duration of fruit development, coordination of ripening changes (climacteric vs. non-climacteric type) and biochemical composition at ripeness is linked to sweetness and acidity. The main constituents of mature fruit result from different strategies of carbon transport and metabolism. Thus, the timing and nature of phloem loading and unloading can largely differ from one species to another. Furthermore, accumulations and transformations of major soluble sugars, organic acids, amino acids, starch and cell walls are very variable among fruit species. Comparing fruit species therefore appears as a valuable way to get a better understanding of metabolism. On the one hand, the comparison of results of studies about species of different botanical families allows pointing the drivers of sugar or organic acid accumulation but this kind of comparison is often hampered by heterogeneous analysis approaches applied in each study and incomplete dataset. On the other hand, cross-species studies remain rare but have brought new insights into key aspects of primary metabolism regulation. In addition, new tools for multi-species comparisons are currently emerging, including meta-analyses or re-use of shared metabolic or genomic data, and comparative metabolic flux or process-based modeling. All these approaches contribute to the identification of the metabolic factors that influence fruit growth and quality, in order to adjust their levels with breeding or cultural practices, with respect to improving fruit traits.

## Introduction

**FIGURE 1:**
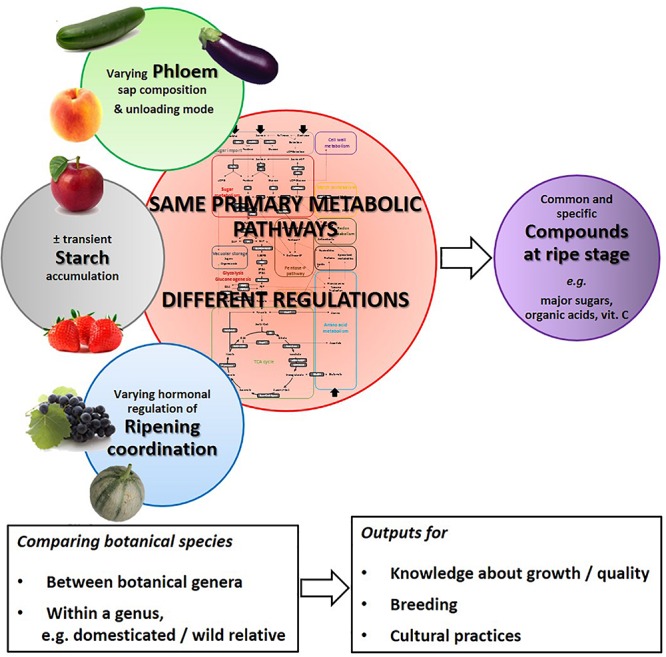
Schema of the major physiological and metabolic differences and similarities between fleshy fruit species leading to varying fruit compositions at ripe stage.

Fresh fruits (866 Mt worldwide in 2016^1^) and their derived products are economically important. Besides their energetic role in human diet linked notably with their carbohydrate contents, they are also crucial for human nutrition and health, especially in relation with their contents in vitamins, anti-oxidants and fibers (Baldet et al., 2014; Rodriguez-Casado, 2016; Wang et al., 2016; Aune et al., 2017; Padayachee et al., 2017). In fruit tissues, primary metabolism can be defined as the biochemical processes that are necessary for growth and development and shared by a large number of taxonomic groups (Verpoorte, 2000), and produces metabolites that are generally essential for organism survival (Aharoni and Galili, 2011). It contributes to flesh growth and ripening, and final fruit quality, particularly sweetness and acidity. Its operation varies according to botanical species and developmental stages. Different botanical species may differ in the composition of the phloem sap originating from leaves and unloaded into the fruit, the occurrence of transient starch accumulation during development, the hormonal orchestration of ripening changes, and the major metabolites accumulated at ripening (Figure 1). All these aspects are linked with primary metabolic pathways involving carbohydrates, organic acids, and amino acids. These pathways are regulated along fruit development that can last from a few dozens to more than 200 days-post-anthesis (DPA), from fruit set to ripe fruit, depending on the species (Table 1). Early development stages after fruit set are usually characterized by a high concentration of organic acids whereas ripening is associated with soluble sugar accumulation (Famiani et al., 2015; Beauvoit et al., 2018). However, the regulation of metabolic pathways is not that simple.

**TABLE 1:** Major characters linked with primary metabolism and differing between temperate fruit species.

**Botanical family**	**Species**	**Fruit development duration (DPA)**	**Climacteric ripening ^a^**	**Major phloem transported sugar^a^**	**Transient starch storage**	**Major soluble sugars in ripe fruit**	**Major organic acids in ripe fruit**	**Major amino acids in ripe fruit**	**References**
Actinidiaceae	Kiwifruit *Actinidia deliciosa Actinidia chinensis*	237	Yes^2^	Sucrose	Yes	Glucose/fructose	Quinate/citrate	Aspartate	Chen et al., 2017; Klages et al., 1998; Nardozza et al., 2013; Richardson et al.,2011
	Hardy kiwifruit (kiwai) *Actinidia arguta*	140	Yes		Yes	Sucrose	Quinate/citrate	–	Kim et al., 2012; Klages et al., 1998; Mikulic-Petkovsek et al.,2012
Cucurbitaceae	Cucumber *Cucumis sativus*	20	No^2^	Raffinose/stachyose/sucrose^4^		Fructose/glucose	Malate (commercial)/citrate (physiological)	–	Hu et al., 2009; McFeeters et al.,1982
	Melon *Cucumis melo*	48	Yes for cantaloupe^2^ No for honeydew^1^	Raffinose/stachyose/sucrose		Sucrose	Citrate	Glutamate/glutamine/GABA	Mitchell et al., 1992; Wang et al.,1996
	Watermelon *Citrullus lanatus*	55	No^2^	Raffinose/stachyose/sucrose^3^		Sucrose	Malate		Gao et al., 2018; Zhang and Ge,2016
Ericaceae	Blueberry *Vaccinium corymbosum*		Yes^2^			Glucose/fructose	Citrate		Mikulic-Petkovsek et al.,2012
Grossulariaceae	Blackcurrant *Ribes nigrum*					Glucose/fructose	Citrate	Glutamine/α-alanine	Burroughs, 1960; Mikulic-Petkovsek et al.,2012
	Redcurrant *Ribes rubrum*					Glucose/fructose	Citrate	Glutamine/α-alanine	Burroughs, 1960; Mikulic-Petkovsek et al.,2012
Rosaceae	Apple *Malus domestica*	160	Yes^2^	Sucrose/sorbitol^4^	Yes	Fructose	Malate	Asparagine/aspartate/glutamate	Brookfield et al., 1997; Drake and Eisele, 1999; Zhang et al.,2010
	Apricot *Prunus armeniaca*	65	Yes^2^	Sucrose/sorbitol^4^		Glucose/fructose (cv. Harcot) sucrose (cvs Bavinity and Trevatt)	Citrate/malate		Bae et al., 2014; Wills et al.,1983
	Blackberry *Rubus fruticosus*		Yes/no			Glucose/fructose	Isocitrate	Asparagine/glutamate	Burdon and Sexton, 1993; Burroughs, 1960; Mikulic-Petkovsek et al., 2012; Whiting,1958
	Sweet cherry *Prunus avium*		No^2^	Sucrose/sorbitol^4^		Glucose/fructose	Malate		Usenik et al., 2008; Wills et al.,1983
	Chokeberry *Aronia melanocarpa*					Sorbitol/glucose	Malate		Mikulic-Petkovsek et al.,2012
	Eastern shadbush *Amelanchier canadensis*					Glucose/fructose/sorbitol	Malate		Mikulic-Petkovsek et al.,2012
	Peach *Prunus persica*	125	Yes^2^	Sucrose/sorbitol^4^	Yes	Sucrose	Malate/citrate	Asparagine/glutamate/proline	Moing et al., 1998; Wills et al.,1983
	Pear *Pyrus communis Pyrus pyrifolia*		Yes^2^	Sucrose/sorbitol^4^	Yes	Fructose	Malate/citrate	Asparagine/serine	Chen et al., 2007; Drake and Eisele, 1999; Mesa et al.,2016
	Prune *Prunus domestica*			Sucrose/sorbitol^4^		Glucose/fructose/sucrose (cultivar dependent)	Malate		Wills et al.,1983
	Plum *Prunus salicina*	91	Yes/no			Glucose/fructose	Quinate		Bae et al.,2014
	Raspberry *Rubus idaeus*		No^2^			Glucose/fructose	Citrate	Serine/α-alanine	Burroughs, 1960; Mikulic-Petkovsek et al.,2012
	Rowanberry *Sorbus aucuparia*					Sorbitol	Malate		Mikulic-Petkovsek et al.,2012
	Strawberry *Fragaria x ananassa*	39	No^2^	Sucrose^3^	Yes	Glucose/fructose or glucose/fructose/sucrose cultivar dependent	Citrate	Asparagine/glutamine	Burroughs, 1960; Mikulic-Petkovsek et al., 2012; Moing et al., 2001; Souleyre et al.,2004
	Wild strawberry *Fragaria vesca*					Glucose/fructose/sucrose	Citrate		Mikulic-Petkovsek et al.,2012
Rutaceae	Clementine/mandarine *Citrus clementina Citrus unshiu*		No^2^		No	Sucrose	Citrate		Legua et al., 2014; Mehouachi et al.,1995
	Acidic lemon *Citrus limon*	150	No^2^			Glucose/sucrose	Citrate		Albertini et al.,2006
	Acidless lemon *Citrus limon*	150	No^2^			Fructose	Citrate/malate/quinate	Glutamate/alanine	Albertini et al., 2006; Brückner and Westhauser,1994
	Acidic lime *Citrus latifolia*	150	No^2^			Glucose/sucrose	Citrate		Albertini et al.,2006
	Acidless lime *Citrus limettioides*	150	No^2^			Fructose	Citrate/malate/quinate		Albertini et al.,2006
	Acidic orange *Citrus sinensis*	150	No^2^			Glucose/fructose	Citrate		Albertini et al.,2006
	Acidless orange *Citrus sinensis*	150	No^2^	Sucrose		Fructose	Quinate	Glutamate/alanine	Albertini et al., 2006; Brückner and Westhauser, 1994; Hijaz and Killiny,2014
Solanaceae	Eggplant *Solanum melongena*		Yes/No^2^			Glucose/fructose	Malate		Kozukue et al., 1978; Makrogianni et al.,2017
	Pepper *Capsicum chilense*	70	Yes/no^1^			Glucose/fructose	Citrate (red) Malate (green)	GABA/proline/asparagine	Flores et al., 2012; Osorio et al.,2012
	Goji berry *Lycium barbarum*					Glucose/fructose	Citrate/malate		Mikulic-Petkovsek et al.,2012
	cultivated tomato, *Solanum lycopersicum*	40–60	Yes^1,2^	Sucrose^4^	Yes	Glucose/fructose	Citrate/malate	Glutamate/glutamine	Schaffer and Petreikov, 1997; Schauer et al.,2004
	Wild tomato, *S. neorickii, S. chmielewskii, S. habrochaites S. pennellii*	40–60				Sucrose	Citrate/malate	Tryptophan (*S. habrochaites*) Aspartate (S. *chmielewskii*) Pyoglutamate/aspartate (*S. neorickii*) GABA (*S. pennellii)*	Schauer et al.,2004
Vitaceae	Grape berry *Vitis vinifera*	100–110	No^2^	Sucrose	Little or no	Glucose/fructose	Malate/tartrate	Proline/alanine/GABA	Dai et al., 2013; Guan et al., 2017; Ollat et al., 2002; Swanson and El-Shishiny,1958

*Fruit development duration in days post-anthesis (DPA), ripening type (climacteric or non-climacteric) and compositional characteristics of phloem sap and ripe fruit. ^*a*^Defined according to Barry and Giovannoni (2007)^1^, Paul et al. (2012)^2^, Rennie and Turgeon (2009)^3^, Fu et al. (2011)^4^.*

Studies dealing with fruit metabolism include biochemical analyses of metabolites from targeted analyses to metabolomics in tissues or sap, measurement of enzyme activity and regulation, transcriptomics, map-based cloning or genome (re)sequencing. Several of these approaches can be combined for one cultivar across fruit development or use a large collection of genetic resources. This review focuses on comparing species for the programming and integration of primary metabolic pathways with growth and fruit quality, mostly for temperate fruits. Such comparisons should help identifying key regulation points, for instance regarding the trade-offs between fruit yield and quality, and possibly propose hypotheses for breeding or agricultural practices.

## Similarities and Dissimilarities Are Noticed in the Composition of Primary Metabolites in Fruits

Fruit taste is strongly influenced by sweetness and acidity, which are associated to sugars and organic acids, respectively. Sugars are abundant in most fleshy fruits; so much that fructose has been named after them (i.e., after the Latin *fructus*). Besides, several amino acids influence the so-called umami taste. The composition and concentrations of these major constituents of the ripe fruit vary according to species (Table 1).

### Soluble Sugars and Organic Acids

Concerning soluble sugars, hexoses are usually more abundant than sucrose. This is the case for most berries, e.g., raspberries, blackberry (Mikulic-Petkovsek et al., 2012) and grape berry (Dai et al., 2013), as well as kiwifruit (Richardson et al., 2011), pepper (Osorio et al., 2012), eggplant (Makrogianni et al., 2017), and cherry (Wills et al., 1983; Usenik et al., 2008). In some species, fructose is more abundant than glucose and sucrose, such as in apple and pear (Drake and Eisele, 1999) or blackcurrant (Mikulic-Petkovsek et al., 2012). However, there are also species in which sucrose is the most abundant sugar, such as mandarin (Legua et al., 2014), peach (Wills et al., 1983), watermelon (Gao et al., 2018), melon (Wang et al., 1996), and hardy kiwi (Klages et al., 1998; Mikulic-Petkovsek et al., 2012). Finally, some species contain sucrose in almost the same proportion as hexoses, for instance several cultivars of litchi (*Litchi chinensis*) (Wang et al., 2006) and cultivated as well as wild strawberry (Moing et al., 2001; Mikulic-Petkovsek et al., 2012).

Sugar alcohols are a major component for some fruit species. Sorbitol, which is common in Rosaceae trees, is present in notable quantities in the developing peach and apricot fruit (Bae et al., 2014). It is also one of the main sugars in ripe chokeberry, rowanberry and eastern shadbush (Mikulic-Petkovsek et al., 2012). Another sugar-alcohol, *myo*-inositol, is present in the early stages of kiwifruit and hardy kiwi development (Klages et al., 1998).

Concerning organic acids, a recent review (Famiani et al., 2015) and research study (Mikulic-Petkovsek et al., 2012) listed the main organic acids found in fruits of more than 50 species. Citrate and malate are the major organic acids in many fruit species. Typically, young fruits are likely to accumulate malate, which will tend to be replaced by citrate at ripening (McFeeters et al., 1982; Flores et al., 2012). Thus, species such as lime, orange, raspberry, strawberry, blueberry, and melon (Wang et al., 1996) accumulate high levels of citrate, while other species such as apple, cherry, chokeberry, rowanberry, eastern shadbush, watermelon (Gao et al., 2018), and eggplant (Kozukue et al., 1978) build up in malate. Other species, e.g., pear, apricot, goji berry, and blackcurrant accumulate both organic acids. In some cases, other organic acids are also overrepresented, as for example isocitrate in blackberry, tartrate in grape berry and lychee, or quinate in kiwifruit and hardy kiwi (Kim et al., 2012).

Large compositional differences for major compounds have been reported between domesticated species and wild relatives. For instance, the tomato domesticated species (*Solanum lycopersicum* L.) accumulates hexoses whereas several wild species (*Solanum neorickii*, *Solanum chmielewskii*, *Solanum habrochaites*) accumulate sucrose as the major sugar (Yelle et al., 1988; Schauer et al., 2004). Furthermore, wild tomato species (*Solanum pennellii*, *S. neorickii*, *S. chmielewskii*, *S. habrochaites*) were found to accumulate higher levels of malate and citrate (Schauer et al., 2004; Steinhauser et al., 2010) than cultivated tomatoes. Similarly, the domestication of mandarin led to a strong decrease in citrate (Wang et al., 2018). Large compositional differences may even be found between cultivars of a given species. An example is given for acidic lemon and lime, where glucose and sucrose are the major sugars, and citrate the main organic acid, whilst in acidless lemon and lime, fructose is the major sugar and citrate, malate and quinate are equally present (Albertini et al., 2006).

### Amino Acids

Regarding amino acids, there are also differences according to botanical species. For berries such as strawberry, glutamine, and asparagine are the most abundant ones whereas blackberry accumulate asparagine and glutamate. For blackcurrant, orange, and lemon glutamine is the major amino acid besides alanine (Burroughs, 1960; Brückner and Westhauser, 1994). The latter one is also abundant in raspberry with serine (Burroughs, 1960). In grape berry, proline, arginine, glutamine and alanine dominate in berry skin, while proline, alanine and γ-aminobutyric acid (GABA) dominate in berry pulp (Guan et al., 2017). For several Rosaceae trees, asparagine dominates with glutamate and aspartate in apple (Zhang et al., 2010), with serine in pear (Chen et al., 2007), and with glutamate and proline in peach (Moing et al., 1998). Concerning Solanaceae, asparagine, GABA, and proline seem to be the predominant amino acids in pepper (Osorio et al., 2012). Glutamine and glutamate are the major ones in domesticated tomatoes (*S. lycopersicum*) together with GABA which is also present in high quantities (Schauer et al., 2004). However, two wild tomato species largely differ according to the latter authors: *S. habrochaites* harbors a high tryptophan content, and *S. pennellii* a high GABA pool, five times higher than in the domesticated species. In kiwifruit, aspartate is the main amino acid (Nardozza et al., 2013).

## Phloem Loading and Unloading Strategies Differ Among Fruits

Fruits are strong sinks attracting plenty of photoassimilates transported from leaves via phloem. From photosynthetic site to sink, photoassimilates need at least three transporting steps, including phloem loading, phloem long-distance transport, and phloem unloading. The strategies of phloem loading, unloading, and the transported forms of carbon are diverse (Rennie and Turgeon, 2009; Braun et al., 2013). Sucrose is the main photoassimilate transported in phloem in most fruit species such as cultivated tomato, grape, sweet orange and cultivated strawberry (Swanson and El-Shishiny, 1958; Rennie and Turgeon, 2009; Fu et al., 2011; Hijaz and Killiny, 2014). However, several fruit species of the Cucurbitaceae family, such as cucumber, watermelon, and melon also transport oligosaccharides including raffinose and stachyose, in higher or equal concentration than sucrose (Mitchell et al., 1992; Rennie and Turgeon, 2009; Fu et al., 2011). Tree species from the Rosaceae family, such as apple, peach, plum or prune, apricot and sweet cherry also transport sugar alcohols (e.g., sorbitol) (Rennie and Turgeon, 2009; Fu et al., 2011). For example, sorbitol can account for 60–90% of the carbon transported in phloem in peach tree (Moing et al., 1997).

### Phloem Loading

Sucrose can be loaded into phloem by three loading strategies, including active apoplasmic, active symplasmic (also called polymer trapping), and passive symplasmic routes (Rennie and Turgeon, 2009; Fu et al., 2011). Active apoplasmic loaders normally have low-abundant plasmodesmata in leaf and require the presence of sucrose transporters (SUTs) and hexose and sucrose transporters (SWEETs), such as in tomato leaf (Fu et al., 2011; Jensen et al., 2016; Liesche and Patrick, 2017). In active symplasmic loaders, sucrose diffuses into the companion cells through abundant plasmodesmata, and is enzymatically converted into sugar oligomers (e.g., raffinose and stachyose), which are molecularly larger and cannot diffuse back to phloem parenchyma, forming a polymer trapping mechanism. Fruit species of Cucurbitaceae family, such as cucumber, watermelon and melon, are active symplasmic loaders (Rennie and Turgeon, 2009; Fu et al., 2011). Passive symplasmic loading requires abundant plasmodesmata to allow sucrose diffusion or convection from mesophyll cells to sieve elements following the sugar concentration gradient. Strawberry is a passive loader, and grape is a candidate passive loader (Rennie and Turgeon, 2009). The phloem loading strategies for sugar-alcohols (e.g., sorbitol, mannitol) can be active apoplasmic or passive symplasmic (Reidel et al., 2009). Most fruit trees of Rosaceae family, including apple, apricot, sweet cherry, peach, and pear are passive symplasmic loaders (Reidel et al., 2009; Fu et al., 2011). Multi-species comparison analysis showed that active loading is associated with efficient water conduction and maximized carbon efficiency and growth, while the reverse is true for passive loading (Fu et al., 2011). A meta-analysis of 41 species with a modeling approach further showed that phloem sugar concentrations are in average at 21.1% for active loaders and 15.4% for passive loaders (Jensen et al., 2013). The theoretical optimum sugar concentration in phloem sap proposed was 23.5%. Organic acids are also transported in phloem (Fiehn, 2003), although references are rare. Amino acids, for instance arginine and glycine in grapevine (Gourieroux et al., 2016), also use phloem as the main transport route from source to sink, and are also transported in xylem sap (Tegeder and Hammes, 2018).

### Phloem Unloading

In fruit sinks, photoassimilates (sucrose, sugar-alcohols, or oligosaccharides) need to be unloaded following symplasmic or apoplasmic pathways (Braun et al., 2013). In apple and cucumber fruits, phloem unloading is apoplasmic throughout fruit development (Zhang et al., 2004; Hu et al., 2011). In several fruits, shifts between the two phloem unloading strategies can occur during development. Tomato and grape fruits operate symplasmic unloading during early development stage when soluble sugar is low, and switches to apoplasmic unloading during fruit ripening when soluble sugars accumulate (Ruan and Patrick, 1995; Patrick, 1997; Zhang et al., 2006). For kiwifruit, Chen et al. (2017) showed that sucrose phloem unloading occurs mainly through the apoplasmic route along fruit development (44–135 days after blooming). However, Gould et al. (2013), working from 22 to 200 days after anthesis, found that phloem unloading dominantly appeared via symplasmic route in early fruit development, while an apoplasmic route becomes important during the later developmental stages. The dominant symplastic import of sugar at the initial stages of fruit development allows a high inflow of carbon input via mass flow. Shifting from symplasmic to apoplasmic unloading during fruit ripening limits back-flow of assimilates from fruit sink to sieve elements and likely facilitates sugar accumulation to high concentrations in fruit tissues (Ruan and Patrick, 1995; Patrick, 1997; Zhang et al., 2006). For amino acid unloading, most plant species follow a symplasmic process driven by a downhill concentration gradient (Tegeder and Hammes, 2018). The question whether and how loading strategies, carbon loading forms, and unloading strategies influence fruit growth and quality are still under debate.

## Primary Metabolism Pathways Are Differentially Regulated

### Sugar and Sugar-Alcohol Metabolism

In most fruits, the main source of carbon is imported via phloem in form of sucrose, which can be degraded via the reactions catalyzed by cell wall invertase in the apoplast, neutral invertase or sucrose synthase following symplastic import into the cytosol, or acid invertase following subsequent import into the vacuole. Carbon import patterns are highly variable from one species to another. For example, in sweet pepper both vacuolar and neutral invertases have been proposed as determining carbon import at young stages (Nielsen et al., 1991). This contrasts with kiwifruit, in which sucrose synthase has been proposed as controlling most of the carbon import in growing fruits (Chen et al., 2017). In the same species, the previous measurement of neutral invertase and sucrose synthase cytosolic enzymes seemed in agreement with symplastic phloem unloading throughout fruit development before ripening (Nardozza et al., 2013). Parietal invertase has been found to impact significantly tomato sugar content at maturity (Fridman et al., 2004). A low level of acid invertase activity and the absence of sucrose synthase activity in *S. chmielewskii*, a wild tomato species, were associated with the high content of sucrose (Yelle et al., 1988). In contrast to *S. lycopersicum*, the capacity of most enzymes of glycolysis and the tricarboxylic acid (TCA) cycle of *S. pennellii*, which also accumulates hexoses, is maintained and increases even during the ripening of the fruit, probably reflecting the fact that the fruit continues to grow until maturity (Steinhauser et al., 2010). For sorbitol-transporting fruit species, imported sorbitol is converted into fructose by sorbitol dehydrogenases (Park et al., 2002). In apple, for instance, fructose is stored in the vacuole or metabolized (Berüter et al., 1997). Sorbitol oxidase (Lo Bianco and Rieger, 2002) and sorbitol-6-phosphate dehydrogenase (S6PDH) (Ohkawa et al., 2008) may also play a role in sorbitol metabolism in Rosaceae fruit trees. For Cucurbitaceae, imported raffinose and stachyose are rapidly metabolized via a pathway that includes enzymes of sugar hydrolysis, phosphorylation, transglycosylation, nucleotide sugar metabolism, sucrose cleavage and synthesis, with an initial implication of α-galactosidases (Dai et al., 2011).

In the cytosol, hexoses resulting from import, degradation or export from the vacuole are phosphorylated via the reactions catalyzed by hexokinases (both hexoses) or fructokinases (fructose only). It has been proposed that the high capacities found for these enzymes in young growing fruits promote high fluxes through glycolysis (Biais et al., 2014). Hexoses phosphates are partitioned between cytosol and plastids, although, unlike leaf plastids, fruit plastids are capable of importing hexoses phosphates (Batz et al., 1995; Butowt et al., 2003). Fructose-6-phosphate is phosphorylated via the reaction catalyzed by ATP- or pyrophosphate-dependent phosphofructokinases (only the former is found in both cytosol and plastid), which enables its breakdown via glycolysis in both compartments. Results obtained in banana suggest that these enzymes are inhibited by phospho*enol*pyruvate (PEP) via allosteric feedback, indicating that there is a crossed glycolytic flux control between PEP and fructose-6-phosphate, which activates PEP carboxylase (Turner and Plaxton, 2003). In both the cytosol and plastid, glucose-6-phosphate tends to equilibrate with fructose-6-phosphate and glucose-1-phosphate via the reaction catalyzed by phosphoglucose isomerase and phosphoglucomutase, respectively, which are present in both compartments. In the cytosol, glucose-1-phosphate is the precursor of uridine diphosphate glucose (UDP-glucose, via the reaction catalyzed by UDP-glucose pyrophosphorylase), precursor of cell wall (Reiter, 2002, 2008; Mohnen, 2008), ascorbate and sucrose (Reiter and Vanzin, 2001). In the chloroplast, glucose-1-phosphate is converted into adenosine diphosphate glucose (ADP-glucose), the precursor of starch. In most fruits, the acquisition of sweetness at maturation is the result of important metabolic changes leading to sugar accumulation (Bonghi and Manganaris, 2012). Of these, starch degradation is often a major source of sugars and energy as detailed below. Finally, sugar vacuolar storage is probably one of the most important, although overlooked, features regarding fruit sweetness. In particular, modeling sugar metabolism in tomato fruit suggested that tonoplastic sucrose and hexose transporters are major control points that condition fruit sugar content (Beauvoit et al., 2014), in line with dramatic alterations in fruit sugar accumulation provoked by the overexpression of a tonoplast transporters in melon (Cheng et al., 2018).

### Organic Acid Metabolism

Malate, citrate, quinate, and tartrate constitute the four main organic acids accumulated to high levels in the vacuoles of fleshy fruits, during their development (DeBolt et al., 2006; Richardson et al., 2011; Tril et al., 2014; Hussain et al., 2017). In fruit, malate is mostly synthetized by the pyruvate kinase bypass, which involves the irreversible carboxylation of phosph*enol*pyruvate into oxaloacetate (OAA) by phosph*enol*pyruvate carboxylase, and OAA is subsequently reduced to malate by cytosolic NAD-dependent malate dehydrogenase (Sweetman et al., 2009; Yao et al., 2011). Citrate is produced from OAA by the TCA pathway, operating in a non-cyclic mode, which is known to take place in plants (Sweetlove et al., 2010) and evidenced in citrus fruits (Katz et al., 2011) with the involvement of mitochondrial citrate synthase (Sadka et al., 2001). Quinate is produced at a branch point of the shikimate biosynthesis pathway by the enzyme quinate dehydrogenase (Marsh et al., 2009; Gritsunov et al., 2018). It is a precursor of chlorogenic acids that are major specialized metabolites in a range of fruit species. Tartrate synthesis results from L-ascorbic acid catabolism through the Smirnoff-Weelher pathway (Melino et al., 2009). L-idonate dehydrogenase, which catalyzes a step in this pathway, is present in grape during the green stage of berry development, concomitantly with the tartrate synthesis peak (DeBolt et al., 2006). Once produced, organic acids are stored into flesh cell vacuoles thanks to an acid trap mechanism, which relies on (i) the existence of a strong pH difference between the cytosol (neutral or slightly alkaline) and the vacuole (highly acidic, pH down to 2.5 in citrus) and (ii) the existence of passive di- and tri-anions transporters on the tonoplast (De Angeli et al., 2013; Etienne et al., 2013). For citrate, the existence of a proton coupled active symporter, CsCit1, has also been reported (Shimada et al., 2006). The regulation of vacuolar malate storage has recently begun to be deciphered (Jia et al., 2018). Once the ripening phase starts, organic acids exit the vacuole and are metabolized to (i) fuel the respiration increase linked to climacteric crisis in climacteric fruits (Colombié et al., 2015) or to meet higher energy demand in non-climacteric fruits such as grapes (Sweetman et al., 2009), or (ii) produce hexoses by neoglucogenesis (Walker et al., 2015; Famiani et al., 2016).

### Amino Acid Metabolism

Amino acid accumulation in developing fruits is the result of both import from phloem and xylem translocation, and *in situ* synthesis (Beshir and Mbong, 2017; Wang L. et al., 2017; Mechthild and Hammes, 2018). Several enzymes of amino acid biosynthesis, including, among others, glutamine synthetase, asparagine synthetase, alanine aminotransferase, and methionine synthase have been detected in global proteomic studies in developing grape berries (Wang G. et al., 2017) or by ^13^C-based flux variance analysis in apple (Beshir and Mbong, 2017). Beside the classical 20 amino acids, fruits can also produce other, non-proteogenic amino acids, such as GABA, which is synthesized through the GABA shunt (Bouché and Fromm, 2004), and possibly β-aminobutyric acid (Thevenet et al., 2017), or citrulline for instance in cucurbits (Fish and Bruton, 2010) including melon (Bernillon et al., 2013) that is produced from arginine (Joshi and Fernie, 2017). Amino acids are not just bricks to build protein in fruits, but also contribute to the global organoleptic qualities of fruits. For example, levels of glutamate contribute to the so-called “umami” taste of tomato (Kurihara, 2015). Amino acid catabolism has been particularly studied in fruits, as it produces numerous quality-related compounds. Phenylalanine leads to the production of polyphenols through the phenylpropanoid pathway, which have antioxidant properties and are health-promoting compounds (Butelli et al., 2008; Cirillo et al., 2014). It is also the starting point of volatile aromas (3-phenylpropanol, 2-phenethylacetate) in melon fruit (Gonda et al., 2018). Isoleucine was shown to be the precursor for 2-methylbutyl ester aromas in strawberry (Pérez et al., 2002) and methoxypyrazines in grape berries (Guillaumie et al., 2013). Thus, amino acid metabolism is a key determinant of fruit quality and palatability.

### Cell Walls and Specialized Metabolites

Fruit primary metabolism also provides building blocks for the synthesis of cell-walls, and non-volatile specialized metabolites (Verpoorte, 2000) besides those mentioned above (e.g., flavonoids, alkaloids, anthocyanins, isoprenoids). Primary cell-wall precursors are mainly supplied as nucleoside diphosphate (NDP) derivatives to produce cellulose, hemicelluloses and pectins (Reiter, 2002, 2008; Mohnen, 2008). Secondary cell-wall lignin precursors, monolignols, are produced by the phenylpropanoid pathway (Zhong and Ye, 2015). Flavonoid and anthocyanin precursors are 4-coumaroyl-coenzyme A (4-coumaroyl-CoA) and malonyl-CoA molecules condensed by chalcone synthase (Jaakola, 2013). Alkaloids are a diverse family of specialized metabolites and are synthesized from various precursors. For instance, steroidal alkaloids of tomato fruit derive from cholesterol (Itkin et al., 2013), whereas tropane alkaloids of deadly nightshade come from arginine and ornithine (Sato et al., 2001). Carotenoids come from both the mevalonic (MVA) and the MVA-independent pathway. Their precursor isopentenyl-diphosphate is either produced from acetyl-CoA or pyruvate and glyceraldehyde-3-phosphate (Fraser and Bramley, 2004). Furthermore, most of these specialized metabolites are decorated with sugars and organic acids. Specialized metabolites have a role in plant defense, but their biosynthesis has a metabolic cost. Thus, allocation theory has been developed to explain resource-based trade-off between plant physiological functions (Bazzaz et al., 1987) and was confirmed experimentally at the plant level (Caretto et al., 2015).

## Starch Does Not Always Accumulate Transiently During Fruit Development

Starch transient accumulation occurs during fruit development in several fleshy fruits such as strawberry, tomato, banana, kiwifruit, apple, and pear. In strawberry, starch accumulates extremely early in the fruit formation process to 3–5% dry weight, and starch degradation predominates thereafter (Moing et al., 2001; Souleyre et al., 2004). In tomato fruit, starch amount peaks at immature green stage, contributing around 20% dry weight (Schaffer and Petreikov, 1997). In apple, starch accumulation occurs continuously from 4 weeks after anthesis until maximal concentration at about 15–17 weeks, then follows a continuous net degradation (Brookfield et al., 1997). In pear, starch degradation starts several weeks before fruit harvest (Mesa et al., 2016). Though kiwifruit and bananas can accumulate more starch than the abovementioned fruit species during fruit growth, nearly 40 and 70% dry weight, respectively, a similar temporal accumulation/degradation pattern is observed (Zhang et al., 2005; Hall et al., 2013; Li and Zhu, 2017). Because of their conserved temporal profiles, starch levels are used to define a ripening index for fruit harvest in several species including apple (Doerflinger et al., 2015). In all these fruits, in addition to a temporal accumulation, starch also shows spatial distribution patterns. In tomato fruit, starch accumulates more in parenchyma (inner pericarp) than in columella (Schaffer and Petreikov, 1997). For different apple cultivars, along with fruit ripening, different spatial starch accumulation/degradation patterns, such as ring or star-shaped pattern, were observed (Szalay et al., 2013). In bananas, starch is lost from the fruit center to the banana outward (Blankenship et al., 1993). Both the temporal and spatial variations of starch in fruits are linked with sucrose-to-starch metabolic enzyme activities (Schaffer and Petreikov, 1997). For example, Shinozaki et al. (2018) showed that the genes encoding enzymes involved in starch biosynthesis, including ADP-glucose pyrophosphorylase (AGPase) and starch-branching enzyme, showed higher expression in parenchyma, which is coherent with the AGPase enzyme activity and starch amount abundance observed in tomato pericarp. Moreover, the AGPase large subunit allele from *S. habrochaites* is characterized by increased AGPase activity in line with higher immature fruit starch content, compared to *S. lycopersicum*. Near-isogenic lines resulting from the interspecific cross of *S. habrochaites* and *S. lycopersicum* allowed showing that the high-starch phenotype was related to a temporal extension of transcription of an AGPase large subunit gene that also conferred higher AGPase activity to the high-starch tomato line (Petreikov et al., 2006, 2009).

Starch plays multiple roles during fruit development. At early fruit set, it is suggested to be a carbon reserve, particularly under mild stress conditions (Ruan et al., 2012). A study on kiwifruit suggested that starch turnover occurs at early developmental stage during cell division (Nardozza et al., 2013). When tomato plants were grown under control, shading or water shortage conditions, fruit hexose and sucrose amounts were similar, but fruit starch contents showed large fluctuations during fruit growth, which suggested that starch may play a buffering role for carbon supply under different abiotic stresses (Biais et al., 2014). Fruit species that do not store carbohydrate reserves such as starch, for instance muskmelon, must remain attached to the plant for the accumulation of soluble sugars to occur during ripening (Hubbard et al., 1990). In fruit species that store starch as a reserve of carbohydrates when fruit is ripening, net starch degradation, attributed to the complex actions of a range of enzymes related to starch breakdown at transcriptional and translational levels in banana (Xiao et al., 2018), also contributes to sugar content in banana (Prabha and Bhagyalakshmi, 1998) or kiwifruit (Nardozza et al., 2013). Petreikov et al. (2009) proposed an increase in transient starch accumulation in tomatoes as a valuable strategy for increasing the sink strength of the developing fruit and its final size and sugar levels. However, starch is not always degraded at fruit maturity. A striking example is the *Musa* genus, where we find dessert bananas characterized by a record degradation of starch (sometimes more than 10% of the dry matter) but also the cooking banana that remains rich in starch at maturity (Hill and Ap Rees, 1994; Jourda et al., 2016).

## Several Cross-Species Studies Highlight Domestication Effects as Well as Mechanisms Shared Across Plant Families

Studies comparing two or more fruit species are usually conducted with species belonging to the same genus or family. They rely on approaches ranging from simple biochemical analysis of metabolites to a combination of omics approaches. The use of introgression lines between a cultivated and a wild fruit species will not be considered in this paragraph, although a range of interesting works contributed to decipher the complexity of sugar or carboxylic acid metabolism, especially in tomato (see Ofner et al., 2016 for a summary of *S. pennellii* introgression lines for instance).

For comparisons within a genus, the parallel study of a cultivated species and one of its close wild relatives may provide insights into the effect of domestication on a primary metabolism pathway and its regulation. For instance, large-scale resequencing of 10 wild and 74 cultivated peach cultivars allowed comparative population genomics that showed an enrichment of gene families related to the carbohydrate metabolic process and TCA cycle within the edible group of peach genotypes (Cao et al., 2014). This work also identified a set of domestication genes, including one encoding a sorbitol-6-phosphate dehydrogenase. The draft genome of peach and whole-genome resequencing of 14 *Prunus* accessions paved the way to comparative and phylogenetic analyses on manually annotated gene families among peach and other sequenced species, and enabled the identification of members with specific roles in peach metabolic processes for instance for sorbitol metabolism, and stressed common features with other Rosaceae species (The International Peach Genome Initiative, Verde et al., 2013).

Regarding another Rosaceae species, apple, a large-scale biochemical study on several hundreds of accessions, revealed that fruits of wild species showed significantly higher level of ascorbic acid than fruits of cultivated species (Fang et al., 2017). Ascorbic acid content was highly positively correlated with malic acid content, but negatively correlated with fruit weight and soluble solid content. As the expression levels of three genes involved in ascorbic acid accumulation were significantly negatively correlated with ascorbic acid contents in fruits, the latter authors suggested a feedback regulation mechanism in ascorbic acid related gene expression. They attributed the differences observed for fruit ascorbic acid content between the wild and cultivated species to an indirect consequence of human selection for increased fruit size and sweetness and decreased acidity.

For tomato, a combination of genome, transcriptome, and metabolome data from several hundreds of genotypes (wild tomato, *S. pimpinellifolium*, *S. lycopersicum* var *cerasiforme*, and *S. lycopersicum* accessions) showed how breeding altered fruit metabolite contents (Zhu et al., 2018). During fruit-size targeted selection, the contents of hundreds of metabolites, including primary metabolites, were changed. The authors propose that the increased primary metabolite content between their big-fruit and their small-fruit accession-pools might be the consequence of a larger metabolic sink in domesticated fruits, and that a range of the related metabolic changes may not be caused by the fruit weight genes themselves but rather be the consequence of linked genes. A study involving *S. pimpinellifolium*, *S. lycopersicum* var *cerasiforme*, and *S. lycopersicum* (Ye et al., 2017) that used a metabolite-based genome-wide association study with linkage mapping and gene functional studies identified a malate transporter (Sl-ALMT9) as being required for malate accumulation during ripening. It also showed that tomato domestication was associated with fixation and extension of favored alleles or mutations that increased malate accumulation.

A comparison of two citrus species, mandarin and orange with a difference in ascorbate content in the pulp (Yang et al., 2011) showed that higher expression of four genes along with lower activity of oxidation enzymes contributes to higher ascorbate in orange. A comparative study of two species of two different genera (Osorio et al., 2012), tomato (climacteric) and pepper (nonclimacteric), based on transcript and metabolite data, unraveled the similarities and differences of the regulatory processes underlying ethylene-mediated signaling in these two fruit types: differences in signaling sensitivity or regulators and activation of a common set of ripening genes influencing metabolic traits.

Finally, an elegant study combining species of three different genera concerns flesh acidity (Cohen et al., 2014). After map-based cloning of *Cucumis melo PH* gene (encoding a membrane protein) from melon, metabolites that changed in a common and consistent manner between high- and low-acid fruits of three species from three different genera, melon, tomato and cucumber, were searched using metabolic profiling. Functional silencing of orthologous *PH* genes in the latter two distantly related botanical families led to fruits with low acidity, revealing that the function of *PH* genes is conserved across plant families.

## New Tools Are Emerging for Multi-Species Comparisons

Inter-species comparisons should not be comparing apples and oranges. In this perspective, an early study highlighted the challenge of aligning the different developmental stages (Klie et al., 2014). It could be partially solved by a more systematic use of development ontologies (Jaiswal et al., 2005) for omics approaches, or by the use of metabolic modeling along development and the cross-species comparison of model topologies and model parameters.

Metabolomics profiling has been used to study fruit metabolism within and between species. Thus, comparison by metabolic profiling of 15 peach cultivars pointed to cultivar-dependent and -independent metabolic changes associated with ripening and to the identification of ripening markers (Monti et al., 2016). The latter authors propose that metabolomics, revealing compositional diversity, will help improve fruit quality. Similarly, the profiling of volatile compounds in nine fruit species revealed that differences were mostly qualitative, with only seven common compounds (Porto-Figueira et al., 2015). Classical multivariate analyses such as principal component analysis (PCA), or more elaborated ones such as STATIS, which handles multiple data tables, are being used to mine metabolite data for comparisons between species. This latter statistical analysis was used at the fruit level to compare five species based on the pattern of 16 primary metabolites, and showed that climacteric species most significantly differed from non-climacteric ones with respect to the metabolism of some sugars and amino acids (Klie et al., 2014). However, tools are still required to take full advantage of the metabolomics datasets describing fruit composition that have been or will be, collected in repositories such as MetaboLights (Haug et al., 2013^2^) or the Metabolomics Workbench.^3^ Although absolute quantitative data are easily reusable and comparable, this is not the gold standard for metabolomics data collected in these repositories, which are generally relative quantification datasets. Normalization methods for appropriate comparison of those data still need to be developed.

The comparative analysis of transcriptomic profiles in varieties of climacteric and non-climacteric melon has highlighted differences, in particular for genes related to ethylene biosynthesis and signaling, but also in gene expression related to sugar metabolism. Indeed, the upward regulation of a soluble (vacuolar) acid invertase could influence the sucrose content of ripe fruit and post-harvest sucrose losses in climacteric fruit, while the upward regulation of invertase inhibitors would explain the high and stable sucrose levels in the non-climacteric variety and could be an important factor in their prolonged shelf-life (Saladié et al., 2015). A comparative study about tomato (climacteric) and pepper (non-climacteric) fruit combined analyses of transcriptomic and metabolic profiles (Osorio et al., 2012). As expected, it showed that genes involved in ethylene biosynthesis were not induced in pepper. However, genes downstream of ethylene perception, such as those implicated in fruit cell wall metabolism or carotenoid biosynthesis, were clearly induced in both Solanaceae species.

For genomics, a computational pipeline has been proposed to identify metabolic enzymes, pathways and gene clusters for about 20 plant species from their sequenced genome including tomato, grapevine and papaya fruit species (Schläpfer et al., 2017). Metabolic pathway databases were generated for 22 species and metabolic gene clusters were identified from 18 species. These vast resources can be used to conduct comparative studies of metabolism regulation between species, with the challenge to decipher organ specificities. Recently, an ambitious study about the evolution of fruit ripening involving transcriptomics, accessible chromatin study and histone and DNA methylation profiling of 11 fruit species revealed three types of transcriptional feedback circuits controlling ethylene-dependent fruit ripening (Lü et al., 2018). Similar approaches could highlight the circuits controlling primary metabolism during fruit growth.

While data on fruit metabolism of different species have been accumulated through years, their use to produce knowledge is now ranging from established statistical approaches to emerging modeling ones (Beauvoit et al., 2018). Modeling approaches involve several tools such as kinetic, stoichiometric or process-based modeling. For tomato, a kinetic metabolic model pointed to the importance of vacuolar storage for sugars (Beauvoit et al., 2014). A stoichiometric model highlighted a climacteric behavior as an emergent property of the metabolic system (Colombié et al., 2015). However, these properties are to be confirmed or infirmed for other fruit species. Recently, process-based models allowed the comparison of sugar concentration in fruits of four species or varieties and showed three species-related modes of sugar concentration control (Dai et al., 2016).

## Conclusion

Although different botanical species share the same primary metabolism pathways, the regulation of these pathways is finely tuned along fruit development in particular ways in different species and results in compositional differences of the ripe fruit (Figure 1 and Table 1). These differences result from genetic and epigenetic modifications linked with evolution, adaptation of species to their environment, domestication or breeding. It seems interesting although challenging, to search whether differences between fruit species for fruit development duration are directly or indirectly related to fruit metabolic characteristics as shown for metabolic profiles and lifespan of yeast mutants (Yoshida et al., 2010), or if differences in maturation duration may be related to mitochondrial metabolism as shown for yeast mitochondrial respiration and redox state and lifespan (Barros et al., 2010). Fruit quality improvement remains one of the major objectives of recent years for breeding. Many tools have been developed to achieve this objective, for instance the use of wild genetic material, omics technology, high-throughput phenotyping or biotechnology (Gascuel et al., 2017). Possible targets to improve sugar levels for instance include adjusting the time of shifting from symplasmic to apoplasmic phloem unloading, modifying sugar vacuolar storage, increasing transient starch storage, or increasing early organic acid accumulation and late neoglucogenesis. Most of the latter targets are linked directly with primary metabolism, but fine regulation networks need further attention. In a comparative study of orange varieties (*Citrus sinensis*), a gene coexpression analysis showed that the sugar/acid ratio-related genes not only encoded enzymes involved in metabolism and transport but also were predicted to be involved in regulatory functions like signaling and transcription (Qiao et al., 2017).

Comparing species helps to identify metabolic factors that influence fruit growth and quality, with a view to manipulating these levels to improve fruit traits. New strategies in species comparison, for instance omics, statistics and modeling, are promising and should continue to be developed in response to the large amount of metabolic data generated by increasingly efficient quantification and identification technologies.

## Author Contributions

All authors listed have made a substantial, direct and intellectual contribution to the work, and approved it for publication.

## Conflict of Interest Statement

The authors declare that the research was conducted in the absence of any commercial or financial relationships that could be construed as a potential conflict of interest.

## Footnotes

1 www.fao.org/faostat

2 https://www.ebi.ac.uk/metabolights/

3 http://www.metabolomicsworkbench.org/
